# Spring-assisted minimally invasive repair of sagittal craniosynostosis

**DOI:** 10.3171/2021.1.FOCVID20103

**Published:** 2021-04-01

**Authors:** Lance S. Governale, Jessica A. Ching

**Affiliations:** 1 Division of Pediatric Neurosurgery, Department of Neurosurgery, and; 2 Division of Plastic and Reconstructive Surgery, Department of Surgery, University of Florida, Gainesville; and; 3 Craniofacial Center, UF Health Shands Children's Hospital, Gainesville, Florida

**Keywords:** craniosynostosis, minimally invasive, cranial spring, pediatric neurosurgery, craniofacial surgery

## Abstract

Craniosynostosis surgery is intended to repair cranial deformity, reduce the risk of increased intracranial pressure from cephalocranial disproportion, and reduce the risk of developmental delays. In recent years, minimally invasive surgical techniques have been developed to achieve these goals with less tissue disruption, lower rates of transfusion, and shorter recovery time. The operation focuses on unlocking the fused bones, while reshaping relies on an adjunct, most commonly a postoperative cranial molding helmet. As an alternative to the care-intensive helmeting process, reshaping with implanted cranial expander springs has emerged. In this video, the authors demonstrate their technique for spring-assisted minimally invasive repair of sagittal craniosynostosis.

The video can be found here: https://vimeo.com/513923721

## Transcript

This case demonstrates our technique for spring-assisted minimally invasive repair of sagittal craniosynostosis. Spring-assisted craniosynostosis surgery was first developed by Dr. Claes Lauritzen in Sweden and later brought to the United States by Dr. Lisa David at Wake Forest.^1,2^

**0:36 Patient Presentation**. The patient is a 3-month-old, otherwise healthy male with no syndrome diagnosis and no family history of craniosynostosis. His exam was notable for scaphocephaly with palpable ridging along the sagittal suture. After a detailed discussion, his family chose spring-assisted minimally invasive repair over the helmet-assisted and open options. We typically perform the spring-assisted surgery between 3 and 6 months of age (corrected for premature birth) underscoring the importance of early patient referral.^2–4^

**1:04 Imaging**. An ultra-low-dose CT scan at 1.1 mGy confirmed sagittal craniosynostosis and patency of the other cranial sutures.

**1:12 Patient Positioning**. The patient is placed prone on gel rolls with the head in the sphinx position. All pressure points are padded.

**1:19 Patient Positioning**. The head is secured in a DORO headrest [Pro Med Instruments]. The endotracheal tube emerges midline, and the ventilation circuit is secured to the headrest system with an elastic tourniquet. The eyes are covered with an occlusive dressing, as is the endotracheal tube tape.

**1:32 Incision Plan**. A small, curvilinear incision approximately 5 cm in length is marked midway between lambda and bregma. The base of the incision is at the midway point. The convexity of the incision is directed posteriorly toward the portion of the suture that is typically more technically challenging. No hair is clipped.

**1:48 Draping**. Adhesive occlusive “thousand” drapes [3M] are placed around a wide prep area to minimize the risk of liquids entering the eyes or loosening the endotracheal tube tape. The skin is prepped with a 2% chlorhexidine/70% isopropyl alcohol ChloraPrep solution [Becton Dickinson], which is allowed to dry for at least 3 minutes. Standard sterile drapes are then applied, including an iodine-impregnated adhesive Ioban drape [3M].

**2:10 Local Anesthesia**. Local anesthesia is provided with infiltration of 0.25% bupivacaine with 1:200,000 epinephrine. Cefazolin is administered as a preincisional antibiotic. Tranexamic acid is given to decrease blood loss.^5^ Cross-matched packed red blood cells are available in the operating room in the rare instance that they are needed.

**2:28 Skin Incision**. A skin incision is made with a No. 15 blade to the subcutaneous adipose layer.

**2:32 Subgaleal**. The incision is deepened to the subgaleal space with monopolar cautery. The scalp flap is elevated in this plane and secured with a suture.

**2:39 Subgaleal Tunnel**. A subgaleal tunnel is created bluntly from the incision to lambda and bregma.

**2:44 Subperiosteal**. Monopolar cautery is used to elevate the periosteum at the intended burr hole site. During the procedure, the periosteum is left intact until shortly before bone removal to decrease blood loss.

**2:53 Drilling Burr Hole**. A burr hole is created with the high speed drill to the epidural space. From this point on, great care is taken to avoid entry into the superior sagittal venous sinus and the subarachnoid space.

**3:04 Widen to 1.5 cm**. The dura is stripped from the bone with an up-angled curet, Woodson elevator, and/or Penfield No. 1. The dura tends to be minimally adherent to the fused suture. Kerrison rongeurs are then used to widen the burr hole to 1.5 cm centered on the skull midline.

**3:17 Kerrison Bone Removal**. The 5-mm Kerrison is then used to extend the 1.5-cm width of bone removal posteriorly being sure to strip the dura before removing bone. The Kerrison is used until the limit of its reach. Hemostasis is achieved at the dura with the bipolar and at the bone edge with bone wax [Ethicon, Johnson & Johnson] and/or absorbable gelatin Gelfoam sponges [Pfizer]. Careful monopolar cautery may also be used. Proceeding expeditiously with bone removal will also help minimize blood loss.

**3:41 Posterior View to Lambdoids**. The lambdoid sutures are identified with certainty. Although they can sometimes be identified extracranially as a white line, the curvature of the bone often prevents this. More typically, they are identified in the epidural space where the dura attaches to the patent sutures. While the endoscope can aid with visualization at the extremes of the exposure, often direct visualization with loupe magnification and headlight illumination is sufficient. Lighted retractors can be helpful as well.

**4:06 Tessier Bone Scissors**. After the limit of the Kerrison's reach, bone removal can continue with the Tessier bone scissors. A bone cut is made along the right and left edge of the 1.5-cm strip.

**4:15 Lambdoid Attachment**. Elevation of the bone strip will show its distal edge attachment at the lambdoid sutures. Using a Penfield No. 1 and an up-angled curet, the bone strip is freed.

**4:23 Free Lambdoid**. The lambdoid sutures are then visible, confirming complete posterior removal of the fused sagittal suture.

**4:29 Posterior Completed**. Here the posterior bone removal is seen from the incision. A temporary strip of Gelfoam is placed along the dura for continued hemostasis and hydration.

**4:36 Anterior Completed**. The 1.5-cm width of bone removal then proceeds anteriorly to the patent coronal sutures or anterior fontanel in a similar fashion.

**4:52 End-On Kerrison**. In addition to the Tessier bone scissors, an end-on Kerrison rongeur [OsteoMed] can sometimes be helpful with bone removal at the extremes of reach. Other options include the drill or an ultrasonic bone cutter.

**5:02 Spring Force Selection**. Two stainless steel cranial expander springs [OsteoMed] are implanted, one directed anteriorly and one directed posteriorly. The spring force is selected based on the patient's age (corrected for premature birth), the thickness of the bone, and the severity of the deformity according to this chart provided by the spring manufacturer.^6^

**5:19 Spring**. The springs are crescent shaped rather than coil shaped. At each end, there is a blunt hook that rests against the bone edge.

**5:25 Spring Placement**. Although the manufacturer provides an optional tool for spring placement, we prefer to place them by hand. The spring is compressed, and the bone edges are engaged by the hooks at the incision. The spring is then advanced posteriorly. In final position, the hooks will contact the bone approximately 1 cm from the patent lambdoid sutures. A notch in the bone can be created at the target contact points, but we do not find this necessary. Care is taken to minimize the number of compressions during placement so as not to alter the force provided by the spring. The force vector of the spring is kept as perpendicular as possible to the axis of the sagittal suture to avoid asymmetrical cranial expansion. For a similar reason, the bone removal is strictly centered on the skull midline.

**6:07 Anterior Spring**. The anterior spring is then placed in a similar fashion. In final position, the hooks will contact the bone approximately 1 cm from the patent coronal sutures. If the bone is very thin, the hook may slightly project into the bone, but this tends to have no adverse effect. The apex of the anterior spring will rest superficial to the apex of the posterior spring.

**6:33 Spring Fixation**. The springs are then secured to the bone at their overlap point on both sides with a 2-0 Vicryl suture [Ethicon, Johnson & Johnson]. The knot is then rotated medially to avoid scalp erosion.

**6:52 Final Anterior View**. Here is the final anterior view. After copious irrigation, a new strip of Gelfoam is placed along the dura. We do not feel that placing bone graft is necessary as the 1.5-cm width of bone removal is sufficient for craniosynostosis repair and tends not to leave long-term bony dehiscences.^7,8^

**6:59 Final Posterior View**. Here is the final posterior view.

**7:04 Skin Closure**. Skin closure then proceeds in standard fashion with interrupted inverted Vicryl suture to the galea and a simple running Monocryl suture [Ethicon, Johnson & Johnson] to the skin. The incision is dressed with bacitracin ointment.

**7:15 Postoperative**. After surgery, the child is observed in the postanesthesia care unit, then overnight on the regular pediatric neurosurgery floor. The intensive care unit is not used. Typically, the child appears well and is ready for discharge the next morning.

**7:28 Spring Removal**. Four months later, the child returns for spring removal surgery. The same patient position may be used, although supine position with the head on a horseshoe headrest can also be used.

**7:37 Opening**. The prior incision is opened. The dissection is carried to the spring apices with monopolar cautery, taking care not to injure the dura or the venous sinus.

**7:57 Spring Dissection**. The cup end of a Penfield No. 1 is then used to dissect distally against the spring to the hooks.

**8:25 Spring Cutter**. The spring cutter is supplied by the spring manufacturer [OsteoMed].

**8:32 Spring Cuts**. First, the spring is grasped at the apex (highlighted by the yellow arrow) with a heavy needle driver. The cutter is then used to section the spring at the transition between the curved apex and the straight arm on both sides (highlighted by the red arrows). The spring apex is then removed. Care is taken to avoid patient or surgical personnel injury by the cut ends of the spring.

**8:54 Spring Removal**. Next, the spring arm is grasped by the heavy needle driver (location highlighted by the yellow arrow). The spring arm is rotated in the appropriate direction to release the hook; then it is removed. Bony overgrowth of the spring is very uncommon.

**9:11 Postoperative**. After surgery, the child is observed in the postanesthesia care unit. Most often, they can be discharged home at this point, although an overnight floor admission is an option.

**9:20 Multidisciplinary Follow-Up**. Follow-up by a multidisciplinary craniofacial team over time is critical to optimizing outcomes. After the initial more frequent postsurgical follow-up, the child is seen at least annually until 7 years of age, when their head is close to its adult size.

## Author Contributions

Primary surgeon: both authors. Assistant surgeon: Ching. Editing and drafting the video and abstract: both authors. Critically revising the work: both authors. Reviewed submitted version of the work: both authors. Approved the final version of the work on behalf of both authors: Governale. Supervision: Governale.
